# Traumatic Right Ventricular Rupture after Blunt Cardiac Injury: CT Diagnosis after False Negative Pericardial Window on FAST Due to Concomitant Pericardial Rupture

**DOI:** 10.5811/cpcem.2016.11.32821

**Published:** 2017-01-23

**Authors:** C. Eric McCoy, Mark I. Langdorf

**Affiliations:** University of California, Irvine, Department of Emergency Medicine, Irvine, California

## CASE

A 93-year-old male presented to a Level I trauma center in hemorrhagic shock after a head-on motor vehicle collision in which he was a restrained driver. Initial vitals were temperature 35°C, BP 100/50 mmHg, HR 63 beats/minute, respirations 28/minute, and oxygen saturation of 93% on 10L. Physical exam was significant for Glasgow Coma Scale 14 (−1 for confusion), decreased breath sounds bilaterally with diffuse chest wall tenderness, a seatbelt sign in the driver-side chest and hip distribution. Cardiac exam was without murmur. Bedside focused assessment with sonography for trauma (FAST) was negative, and chest radiograph revealed multiple bilateral rib fractures and large right hemothorax. The patient developed worsening hypotension after a chest tube yielded >1L of gross blood despite blood product resuscitation. Computed tomography of the chest revealed multiple thoracic injuries including but not limited to several rib fractures, sternum fracture, large right-sided hemothorax and right ventricular rupture with leak of contrast and associated hemopericardium (Image). The patient survived an emergent thoracotomy in the emergency department for repair of the right ventricular rupture.

## DISCUSSION

Blunt cardiac injury encompasses multiple injuries, including contusion, acute valvular disorders, and chamber rupture.^1^ Blunt traumatic cardiac rupture is a very rare occurrence accounting for 0.5% of blunt trauma cases with a high mortality rate.^2^ We believe the initial negative FAST scan was due to a concomitant cardiac and pericardial rupture, which allowed blood to drain into the right hemithorax. Coexisting pericardial rupture in patients with cardiac rupture obscures the diagnosis and contributes to mortality.^3^,^4^ False negative pericardial ultrasound secondary to a concomitant pericardial laceration and subsequent decompression of the cardiac hemorrhage into the ipsilateral pleural space is extremely rare and has only been recently described in the literature.^5^ This case/image highlights the importance of considering an underlying cardiac injury in the presence of a negative FAST pericardial window in patients with a traumatic hemothorax.

## Figures and Tables

**Image:**
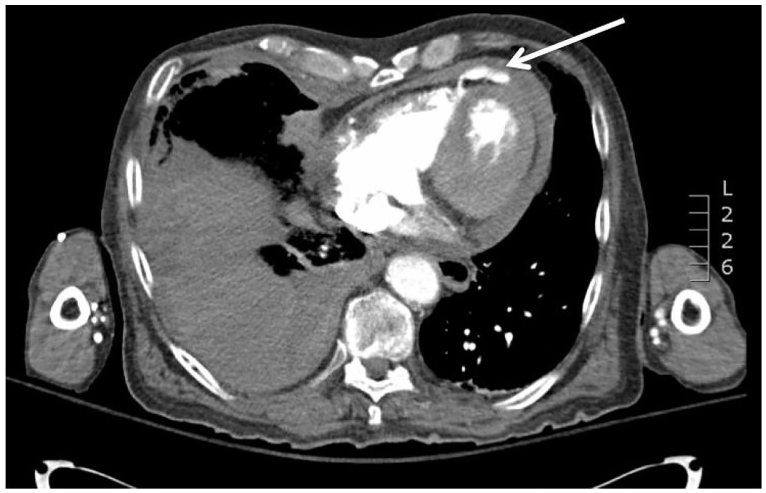
Computed tomography of chest revealing rupture of right ventricle with leak of contrast and associated hemopericardium (white arrow).
